# Daptomycin: A Novel Macrocyclic Antibiotic as a Chiral Selector in an Organic Polymer Monolithic Capillary for the Enantioselective Analysis of a Set of Pharmaceuticals

**DOI:** 10.3390/molecules26123527

**Published:** 2021-06-09

**Authors:** Ali Fouad, Adel A. Marzouk, Montaser Sh. A. Shaykoon, Samy M. Ibrahim, Sobhy M. El-Adl, Ashraf Ghanem

**Affiliations:** 1 Chirality Program, Faculty of Science and Technology, University of Canberra, Bruce, Canberra 2601, Australia; alifouad247@gmail.com; 2 Pharmaceutical Chemistry Department, Faculty of Pharmacy, Al-Azhar University, Assiut 71524, Egypt; Adelmarzouk77@gmail.com (A.A.M.); monoceutical@yahoo.com (M.S.A.S.); 3 Pharmaceutical Chemistry Department, Faculty of Pharmacy, Zagazig University, Zagazig 44519, Egypt; dr-samy2010@hotmail.com (S.M.I.); sobhyeladl@yahoo.com (S.M.E.-A.)

**Keywords:** daptomycin, monoliths, enantioselective, nano-HPLC

## Abstract

Daptomycin, a macrocyclic antibiotic, is here used as a new chiral selector in preparation of chiral stationary phase (CSP) in a recently prepared polymer monolithic capillary. The latter is prepared using the copolymerization of the monomers glycidyl methacrylate (GMA) and ethylene glycol dimethacrylate (EGDMA) in the presence of daptomycin in water. Under reversed phase conditions (RP), the prepared capillaries were tested for the enantioselective nanoliquid chromatographic separation of fifty of the racemic drugs of different pharmacological groups, such as adrenergic blockers, H1-blockers, NSAIDs, antifungal drugs, and others. Baseline separation was attained for many drugs under RP-HPLC. Daptomycin expands the horizon of chiral selectors in HPLC.

## 1. Introduction

Macrocyclic drugs, including ansamycins, glycopeptides, and the polypeptide antibiotic thiostrepton, have been widely utilized as chiral stationary phases (CSPs) for the enantioselective separations of racemates [1,2,3]. In particular, chiral selectors belonging to glycopeptides, such as vancomycin, colistin sulfate, norvancomycin, ristocetin A, teicoplanin, eremomycin, and others, have been successfully used in different chromatographic setups [1,4,5] to achieve the enantioselective resolution of racemic amino acid derivatives and profens [6,7,8,9,10].

Many of these antibiotics CSPs are widely maneuvered in conventional columns. Compared to the well-known vancomycin CSP, eremomycin has trinuclear amino acid fragments in addition to only one chloro-substituted aromatic ring, while vancomycin has two [11]. Such unique features relying on the different binding forms by multifunctional groups supported their use in enantiomeric resolution of a wide range of racemic drugs in CE, HPLC, and CEC [12,13,14,15,16,17,18].

**Figure 1:**
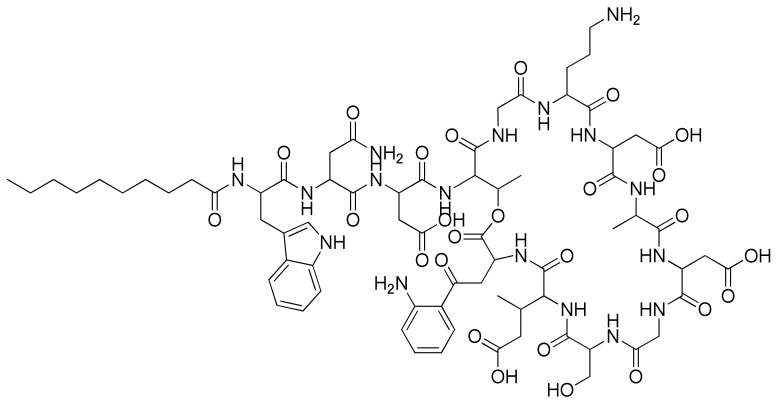
Chemical structure of daptomycin.

Similarly, daptomycin (Figure 1) offers comparable speculation for its possible use in chiral separation. Thus, daptomycin is a cyclic lipopeptide produced by *Streptomyces roseosporus*. It includes ten amino acids, arranged in a cyclic fashion, and three on an exocyclic tail [19,20]. Daptomycin antibiotic acts by disrupting multiple steps of bacterial cell membrane function. It clusters in a phosphatidylglycerol-dependent fashion to alter the curvature of the cell membrane, which forms holes that leak ions and leads to rapid depolarization. Thus, the loss of membrane potential leads to the inhibition of protein, DNA, and RNA synthesis, then apoptosis [21]. Daptomycin is endorsed in adults for skin infections caused by Gram-positive bacteria [22].

In this figure, we investigate the application of daptomycin as a novel chiral selector immobilized and/or encapsulated in organic polymer monoliths for the enantioselective nano-HPLC resolution of a set of racemic drugs.

## 2. Results

### 2.1. Preparation and Characterization of the Monoliths

#### 2.1.1. Preparation of the Monoliths

Macrocyclic antibiotics were previously reported in conventional and capillary enantioselective chromatography for their use as chiral selectors. Moreover, macrocyclics-based silica monoliths have been previously investigated [1,23,24,25,26,27,28,29]. However, the macrocyclic antibiotics were immobilized on the functionalized monoliths by a labor-intensive reductive amination process [25]. In this article, we report a recent application of daptomycin macrocyclic antibiotic immobilized and encapsulated in situ organic polymer monolithic capillaries for enantioselective nano-HPLC (Figure 1). The solubility and miscibility of daptomycin was tested with the solvents used to prepare monoliths. 1,4-butandiol and propanol were used as porogenic solvents because of their formation of a highly homogeneous solution.

**Figure 2:**
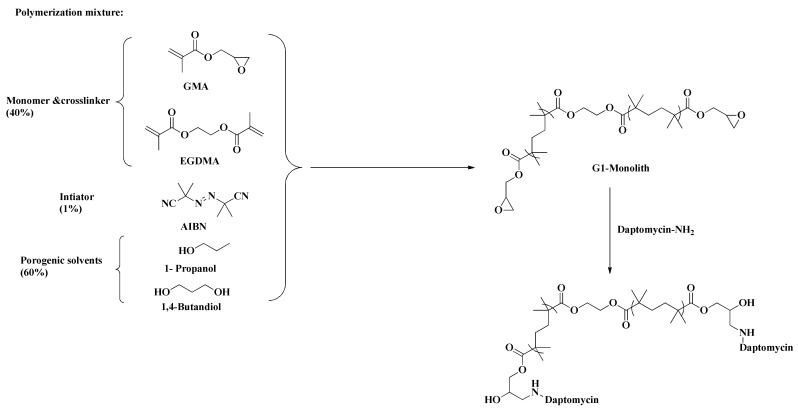
Schematic diagram showing the polymer composition of D2 monolith.

Briefly, a solution of daptomycin in methanol was pumped through the capillaries for 24 h (D1) and 48 h (D2). Under this condition, the active amino group of daptomycin reacts with the highly active epoxy groups of the polymer surface leading to the covalent bonding of daptomycin to the monoliths (Figure 2). To ensure that there would be enough daptomycin to cover the epoxy surface groups, the daptomycin was injected over a two-day period. The polymer used in the immobilization of daptomycin onto the monoliths was previously reported and achieved reproducible capillaries with good properties [30]. To immobilize daptomycin, a simple and effective epoxy method was selected, which also prevents the production of undesirable byproducts [31].

Daptomycin polymer monoliths (D3) were primed via in situ copolymerization of daptomycin with a functional monomer (GMA), a cross linker (EGDMA), a porogenic system (1-propanol, 1,4-butandiol), and daptomycin (6%). The ratio of the monomers to the solvents was 40:60 *w/w*, respectively, to result in reproducible monoliths.

#### 2.1.2. Imaging by SEM and Studying the Surface Characteristics of the Monoliths

**Figure 3:**
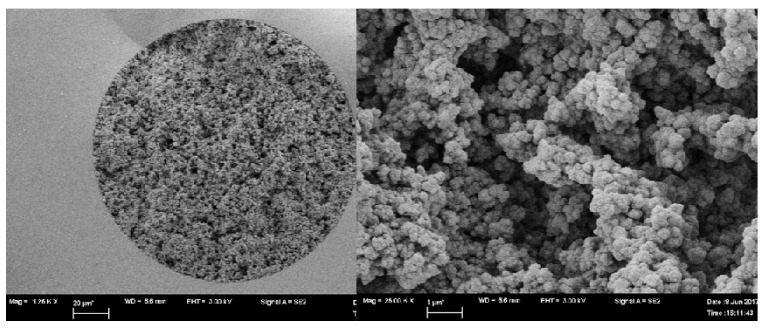
SEM of column D2 at 1200× and 25,000× (left and right, respectively) showing small microglobules with rough surface.

**Figure 4:**
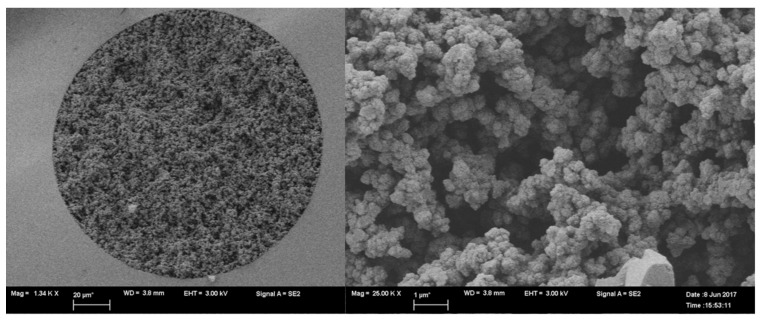
SEM of column D3 at 1200× and 25,000× (left and right, respectively) showing small microglobules with rough surface.

D2 and D3 monoliths were imaged using SEM and exhibited homogenous porous structures with interconnecting canals permitting the flow of solvents under reduced capillary backpressure (Figure 3 and Figure 4). Both capillaries exhibited good linkage to the inner wall in the immobilization or encapsulation of daptomycin in situ organic polymer. The specific surface area and the pore structure of the monoliths were previously studied by our group [32].

#### 2.1.3. Calculation of Total Porosity

The quality of the daptomycin monoliths was measured as a function of the capillary total porosity (ε*_T_*). This gives a partial characterization for the monolith [33,34]. Total porosity was evaluated by the HPLC flow method using uracil as an unretained marker and methanol as the mobile phase [35]. The total porosity was calculated by the following equation:

(1) $$\varepsilon_{T}=\frac{V}{\pi r^{2}u}\times 100$$

where ε*_T_* is the total porosity, V (m^3^/sec) is the solvent volume, r (m) is the inner radius of the capillary, and u (m/sec) is the linear velocity of the mobile phase. Calculated ε*_T_* values for the D1, D2, and D3 are listed in Table 1.

From Table 1, it was observed that ε_T_ column porosity values were lower than expected values, which should be equal or close to a fixed weight of 60% porogens, however higher than our previously prepared columns of amylose and colistin sulfate [1,23]. Since the solvent content composition was kept constant, ε_T_ was supposed to be equal or close to the porogens volume fraction (60%); however, the monolith’s porosity is strongly related to the polymerization conditions, i.e., the content of the polymer, reaction time, temperature, polarity, and concentration of the monomer. In the reaction [36], they affect the early steps of phase separation and have a high impact on the formed monolith [37].

It was also observed that using a high concentration of the chiral selector resulted in a column with low efficiency. This may be due to its accumulation of lumps at a high concentration inside the capillary. The most appropriate concentration of the daptomycin in the mixture was chosen from four different D3 columns with different concentrations of daptomycin at 10, 20, 30, and 40 mg/mL. The measurement of 10 mg/mL resulted in better separation and resolution.

Elemental analysis was done to determine the excess of nitrogen percentage in the capillaries in comparison to a blank column (G column) to ensure the presence of daptomycin in the prepared capillaries. The resulting nitrogen contents are listed in Table 2.

#### 2.1.4. Mechanical Stability

**Figure 5:**
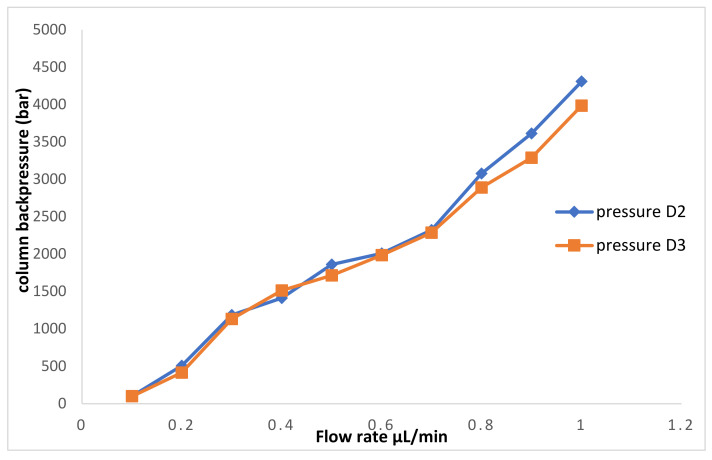
Column backpressure versus mobile phase flow rate of daptomycin monolithic columns, mobile phase: methanol/water 80:20 (*v/v*).

The prepared D2 and D3 columns were evaluated to ensure their mechanical stability using a mixture of methanol/water (80:20 *v/v*) as the mobile phase at a flow rate of (0.1–1 µL/min). The decrease in pressure as a function of flow rate was linear (Figure 5). Furthermore, the tested capillaries showed high stability across the pressure ranges.

### 2.2. Enantioseparation of Racemic Pharmaceutical Drugs

Daptomycin-based monolithic capillaries were tested for the enantioselective nano HPLC resolution of fifty of the racemic drugs of different pharmacological groups, such as adrenergic blockers, NSAIDs, antifungal drugs, norepinephrine-dopamine reuptake inhibitors, neurotransmitters, cCNS depressants, H1-blockers, antibiotics, antimetabolites, antiarrhythmic drugs, and other miscellaneous drugs. For the immobilized daptomycin columns D1 and D2, the first mobile phase selected for the chiral resolution of racemates 1–50 (Supplementary Materials Figure S1) was methanol/water ranging from 95:5 to 5:95 *v*/*v*; few racemates were poorly enantioseparated. Therefore, a mobile phase composed of acetonitrile/water ranging from 95:5 to 5:95 *v*/*v* at a 1 µL/min flow rate and a fixed UV detection of 219 nm was tried.

**Figure 6:**
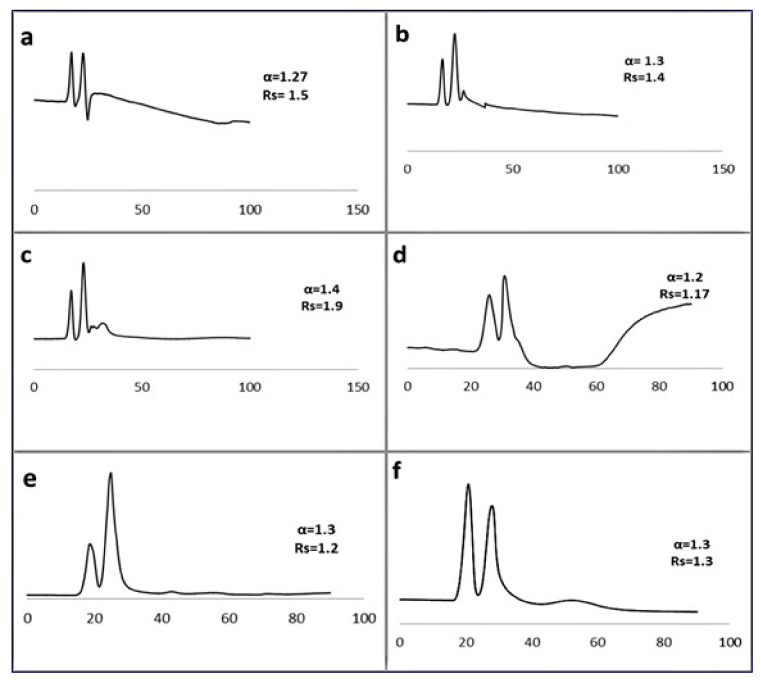
Enantioselective nano-lc separation of racemic Naftopidil (**a**), flavanone (**b**), pindolol (**c**) (Mobile phase: ACN/water 10:90 *v/v*,), Atenolol (**d**), Ampicillin (**e**), and 4-hydroxy-3-methoxymandelic acid 35 (**f**) (Mobile phase: ACN/water 5:95 *v/v* on D2 capillary column (150 µm ID, 25 cm length)). UV: 219 nm, flow rate: 1 µL/min.

The immobilized daptomycin phases D1 did not sequel in any significant separation of the tested racemic samples under various HPLC conditions. However, the second immobilized D2 column resulted in acceptable separation (Rs ≥ 1) using acetonitrile/water from 95:5 to 5:95 *v*/*v* at 1 µL/min for 14 racemates (Figure 6), including fluribiprofen, pindolol, atenolol, naftopidil, 6 hydroxyflavanone, trifloroethanol, flavanone, ampicillin, ibuprofen, ketoprofen, aminoglutethimide, acebutolol, normatenphrine, and 4-hydroxymandelic acid. All chromatographic data for D2 separations is shown in Table 3.

**Figure 7:**
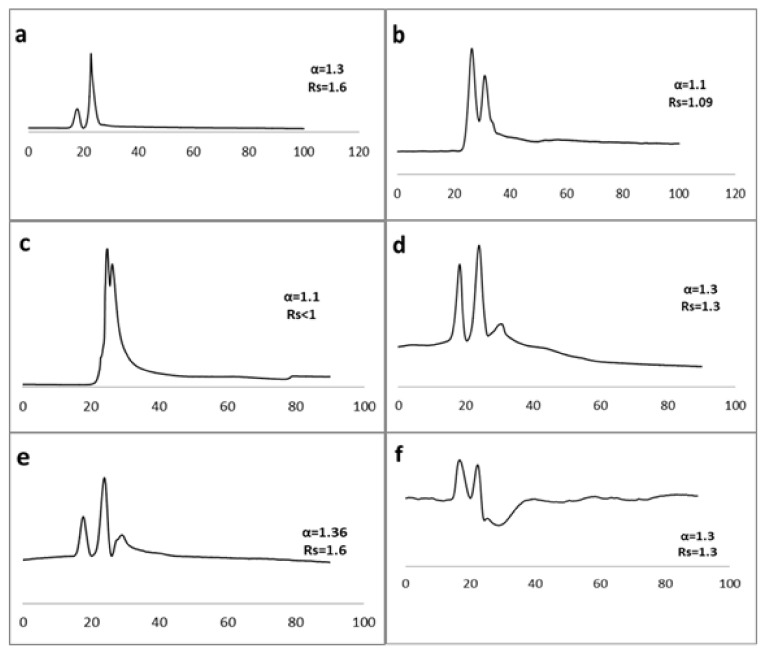
Enantioselective nano-lc separation of racemic Acebutolol (**a**) (Mobile phase: ACN/water 10:90 *v/v*,), pindolol (**b**) (Mobile phase: ACN/water 20:80 *v/v*,), Metoprolol (**c**), pindolol (**d**), 1-Indanol (**e**), and Thalidomide (**f**) (Mobile phase: ACN/water 5:95 *v/v* on D3 capillary column (150 µm ID, 25 cm length)). UV: 219 nm, flow rate: 1 µL/min.

On the other hand, the encapsulated daptomycin chiral capillary D3 provided acceptable separation with many compounds (Rs ≥ 1) (Table 4) using both methanol and acetonitrile mobile phases in different concentrations with water. For examples, in MeOH/H_2_O, from 95:5 to 5:95 *v*/*v*, hexaconazole, aminoglutethimide, *O*-methoxy mandelic acid, and metoprolol were significantly separated (Rs ≥ 1), while in acetonitrile/water checked from 95:5 to 5:95 *v*/*v*, fluribiprofen, propranolol, flavanone, fenprofen, carprofen, pindolol, atenolol, naftopidil, 6 hydroxy flavanone, normatenphrine, indoprofen, tocainide, metoprolol, acebutolol, ibuprofen, 1-Indanol, 4-hydroxymandelic acid, cizoliritin citrate, ampicillin, ketoprofen, and thalidomide were separated. The addition of trifloroacetic acid (TFA) 1% in water (*v*/*v*) as an additive resulted in the separation of few racemates without any additional benefits (Figure 7). Our effort to use NP-HPLC as an *n*-hexane/2-propanol mixture ranging from 10–90% (*v/v*) resulted in the separation of less than one. All chromatographic data are shown in Table 4.

As daptomycin has not been previously tested in chiral resolution, confirmatory tests were performed by injecting the studied separated racemic drugs using a monolithic capillary, although without the addition of daptomycin (blank column) and injecting a single enantiomer on the D3 column. The injected drugs on an untreated column resulted in single peaks under similar chromatographic conditions (Supplementary Materials Figure S2). Although the chiral selector might be soluble in water, the resolution of the tested drugs was mostly achieved with the mobile phase with a high water percentage; therefore, as previously reported, the solvent used for solubilizing the selector can be used as the mobile phase in the same capillary [25]. The comparison of the enatioselective separation of enantiomers using the blank capillary and D3 capillary ensures the presence of daptomycin within the capillary and excludes the dissolution in mobile phase. Furthermore, the achieved repeatability of the D3 capillary confirms the stability of the daptomycin within the functionalized polymer in situ the capillary. The elemental analysis results of nitrogen content also confirm the presence of daptomycin in the capillaries.

The macrocyclic antibiotic family is rich in different functional groups, which gives them the unique characteristics of recognition interactions with racemic analyses in chiral chromatography [27]. Daptomycin is one of these families used recently in chiral chromatography as a chiral selector. Furthermore, the immobilization of macrocylics in organic polymer monoliths in nano-HPLC has not been previously studied. The organic polymer monolith offers advantages over other monolithic columns, rendering it affordable and easily accessed [38]. The conversion from conventional HPLC (mL flow, more solvent) to micro/nano-HPLC (less solvent) resulted in efficient sample preparation and separation technologies, which agreed with the concept of green chemistry. Furthermore, some featured benefits include improved and faster resolutions, low amounts of mobile phase, and better robustness. It is worth mentioning that very low sample volumes lead to reduced sensitivity. However, this problem can be resolved by using more sensitive detectors (e.g., MS).

### 2.3. Columns Repeatability

**Figure 8:**
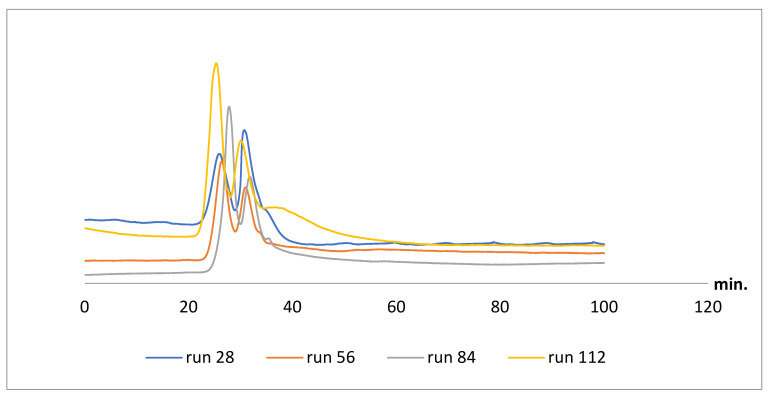
The loadability of the monolithic columns of pindolol started at run number 28 up to run number 112, mobile phase: ACN/water 20:80 *v/v*, UV: 219 nm, flow rate: 1 µL/min.

To examine the repeatability of daptomycin capillaries, two from the encapsulated D3 capillaries were functioned under the same condition on the same day using the same technique to test column-to-column repeatability. Additionally, batch-to-batch repeatability was checked by functioning three different batches at different days using the same polymer mixtures. Pindolol was chosen to test D3 capillaries performance as a function of repeatability, as it was nearly baseline separated on both capillaries. The reproducibility of the retention times of both pindolol peaks was accepted. The capillary run-to-run repeatability average retention times were 21.5 min (RSD = 0.4 %) and 31.3 min (RSD = 0.5 %) for peak one and peak two, respectively. The capillary column-to-column repeatability average retention times were 21.85 min (RSD = 1.6 %) and 31.55 min (RSD = 1.2%) for peak one and peak two, respectively. the capillary batch-to-batch repeatability average retention times were 21.75 min (RSD = 1 %) and 31.65 min (RSD = 1.6 %) for peak one and peak two, respectively. The retention time R*t*s and their relative standard deviation RSD ranged from 0.4% to 1.6%. The results propose the reproducibility of using the daptomycin monolithic capillary for enantioseparation. Additionally, the capillary loadability was checked by running more than 150 runs on the same column; pindolol was run in different orders starting at run number 28 and ending by run number 112. Similar resolution was achieved (Figure 8).

## 3. Discussion

Many studies on macrocyclic antibiotics as chiral selectors in CSPs have been previously reported using true binding mechanisms between the chiral selector and the stationary phase. Thus, immobilization, coating, or covalent bonding have been used to expand the use of different mobile phases and afford a more robust and stable CSP [24,28,39]. Most of the CSPs have been formed by immobilization processes to attach the chiral selectors on the solid supports. However, long-time pumping offers coverage of the CS less than the one pot technique [40].

Such unique features, such as multichiral points, inclusion pockets, aromatic rings, several hydrogen bonding sites, sugar parts, and other polar and nonpolar groups, are the main causes for the usage of macrocyclics as enantioselectors with better chiral resolution abilities in different modes of HPLC and CEC [41,42,43,44,45,46]. Here, the chiral recognition mechanism is heavily dependent on both electrostatic bindings and secondary binding forces, such as hydrogen bonding, complexation, inclusion complexation, steric interactions, dipole interactions, and anionic and cationic binding.

It is well known that the presence of various function groups facilitates more points of binding between the racemic analytes and the CSP and increases the selectivity towards some analytes.

The hydrophobic interactions were predominantly CSP analyte interactions, while hydrogen bonding has important role in the enantioselections between the other racemics and CSPs. Firstly, by testing the D1 capillary with the mobile phase based on methanol or acetonitrile, enantioselective resolution was achieved for few analytes; on the other hand, enantioselective resolution was observed for many analytes on both immobilized and encapsulated columns using acetonitrile/water (D2 and D3). These proved that the amount of daptomycin immobilized in column D1 may be inadequate, or the binding process was not completed. Additionally, this confirms the role of solvent polarity in the enantioselective resolution mechanism in terms of the inclusion complex stability. The use of additives as triflouroacetic acid was used and did not obtain any improved resolution; additionally, they have a negative effect on the age of the capillary and have potential problems with nano-HPLC systems as precipitation into the pumps and valves.

The enantioselective resolution was mostly achieved over a wide range of water content of the mobile phase; this is because water enhances the binding forces between the CSP and the analytes. We assumed that the enantioselective resolution in this part was mainly due to the formation of inclusion complexes as discussed before. Some analytes could not be well separated on these capillaries in n-HPLC. These proved that the amount of daptomycin entrapped in the polymer monoliths or immobilized on it may lead to different chromatographic characters of the capillaries, such as the retention times, resolution, and efficiency.

## 4. Experimental

### 4.1. Reagents and Materials

Daptomycin (99%), ethylene glycol dimethacrylate (EGDMA, 98%), glycidyl methacrylate (GMA, 98%), 1-propanol (99%), 1,4-butanediol (99%), trifluoroacetic acid (TFA, ≥99.5%), sodium hydroxide, and hydrochloric acid were purchased from Sigma-Aldrich (Milwaukee, WI, USA). Ethanol (HPLC grade) and acetone (AR grade) were purchased from BDH (Kilsyth, Victoria, Australia). Methanol (HPLC grade) was purchased from Scharlau (Sentmenat, Spain). All other reagents were of the highest available grade and used as received. The fused-silica capillaries (150 µm internal diameter and 365 µm outer diameter) were purchased from Polymicro Technologies (Phoenix, AZ, USA). 2, 2-Azobis (isobutyronitrile) (AIBN) was obtained from Wako (Osaka, Japan). The enantiomers were mostly purchased from Sigma-Aldrich. The purified water used for experiments was purified by the Nanopure Infinity water system (Thermo, NJ, USA).

### 4.2. Functioning and Characterization of the Monolithic Capillaries

#### 4.2.1. Activation of the Empty Fused Silica Capillaries

Surface activation infused silica capillaries were prepared according to our previously published procedure [23]. First, the fused silica capillaries were washed by acetone 2–3 times, then by water 2–3 times, filled with 0.2 mol/L sodium hydroxide (NaOH) for about 6 h, rinsed with water until reaching pH 7, injected with 0.2 mol/L HCl for 12 h, and washed by ethanol 2–3 times and then by water 2–3 times. Next, a 20% (*w/w*) solution of 3–(trimethoxysilyl) propyl methacrylatedissolved in 95% ethanol with pH 5 adjusted by acetic acid was pumped with a flow rate of 0.25 µL/min for about 6 h. The capillary was then pumped with acetone for washing and dried using a stream of N_2_ for 2–3 min. Lastly, it was left out for one day at room temperature.

#### 4.2.2. Preparation of Immobilized Daptomycin-Based Monolithic Capillary

Two GMA-based monolithic capillaries were arranged according to our previously published procedure without the addition of any chiral selector [23]. For both columns D1 and D2, the polymerization mixture was composed of monomers (20% GMA), 10% cross-linker (10% EGDMA), and 60% porogenic solvents, including 1-propanol 1,4 butandiol and water in percentages of 48%, 6%, and 6%, respectively; all percentages were in *w/w*. AIBN in 1% *w/w* of the monomers was used as a radical initiator for the copolymerization reaction (Figure 2). After the polymerization of the monoliths inside the capillaries and washing them with methanol, a 10 mg/mL solution of daptomycin in methanol was pumped on the epoxy monoliths at 250 nl/min for 24 h and 48 h to obtain D1 and D2 capillaries, respectively. The capillaries were then washed with absolute methanol for 1 h, conditioned with the selected mobile phase for initial testing for 1–2 days, and connected to the nano-HPLC, and the enantioselective analysis was conducted [24].

#### 4.2.3. Preparation of Daptomycin Functionalized Monomer (Encapsulation)

**Figure 9:**
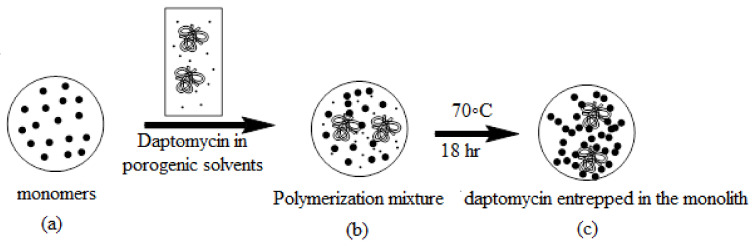
Schematic diagram showing the encapsulation technique of daptomycin.

The monolithic capillary column D3 was prepared according to our previously published procedure [24]. The polymerization mixture was made up of monomers (20% GMA), 10% cross-linker (10% EGDMA), and 60% porogenic solvents, including 1-propanol 1,4 butandiol and daptomycin in percentages of 48%, 6%, and 6% *w/w*, respectively; AIBN in 1% *w/w* of the monomers was used as a radical initiator for the copolymerization reaction. The procedure was completed as previously discussed (Figure 9) [1].

#### 4.2.4. Imaging the Prepared Monoliths by SEM

D2 and D3 monoliths were imaged by scanning electron microscopy (SEM) to study the morphology of the prepared capillaries. The capillaries were prepared for imaging according to our previously published procedure. First, they were cut into ~1 cm parts and put perpendicularly on a 12.7 mm pin-type aluminum stub by double face epoxy resin tape. Then, the SEM imaging was processed, and high-resolution images were collected by sputter staining the capillaries’ parts with gold.

### 4.3. Preparation of Stock Solutions and Samples

Stock solutions of the racemic drugs at concentrations of 1 mg/mL in filtered HPLC grade methanol were prepared. Before injection, these solutions were further diluted ten times and filtered through Sartorius Minisart RC 15 0.2 µm pore size filters (Sartorius AG, Goettingen, Germany). The chemical structures of 50 tested drugs are illustrated in the Supplementary Materials Figure S1 [1,23].

### 4.4. HPLC Conditions

The mobile phase of the mixture contained water/methanol (*v*/*v*) or acetonitrile/water (*v*/*v*) for RP-HPLC and *n*-hexane/2-propanol for NP-HPLC. For all tested samples, the injected volume was 1 µL. Moreover, preparatory UV analyses were performed at a wavelength of 219 nm.

## 5. Conclusions

Daptomycin was used as a novel chiral selector in enantioselective chromatography. It was used to prepare a monolithic capillary based on an organic polymer. The capillaries have been easily prepared by immobilizing or encapsulating daptomycin with the organic polymer and investigated for enantioseperation of chiral drugs. D1 (immobilized) column did not achieve any acceptable separation under both reversed and normal phases. Baseline separation was achieved for many drugs under RP-HPLC conditions using D2 and D3 (immobilized and encapsulated), which were never used in chiral chromatography, while NP-HPLC conditions did not obtain any agreeable separations with any capillary. The method gives a more economical analysis under environmentally benign RP-HPLC conditions.

## Figures and Tables

**Table 1:** Porosity and performance of the prepared polymer monoliths using uracil, mobile phase: methanol 100%, flow rate: 1 μL/min.

Column	ε_T_ (%)
D1	21.75 ± (1.6)
D2	31.25 ± (1.13)
D3	33.1 ± (1.07)

**Table 2:** Measured nitrogen content in different columns.

Column	Nitrogen (% *w/w*)
G	1.8
D3	2.15

**Table 3:** Chromatographic parameters, separation, and resolution factors for the significantly resolved compounds on D2 (immobilized) column, using reversed mobile phases, flow rate: 1 µL/min.

Column D2				
Phase	Mobile Phase	Drug	Separation Factor (α)	Resolution (Rs)
Reversed Phase	ACN:water 35:65	Fluribiprofen 21	1.4	1.8
		Trifloroethanol 41	1.3	1.1
		Pindolol 7	1.3	1.45
		Atenolol 3	1.1	<1
		Naftopidil 8	1.3	1.3
		Ampicillin 25	1.1	<1
		6 hydroxy flavanone 38	1.2	<1
	ACN:water 20:80	Trifloroethanol 41	1.37	1.5
		Flavanone 39	1.3	1.3
		Atenolol 3	1.1	<1
		Ampicillin 25	1.3	1.1
		Pindolol 7	1.1	1
	ACN:water 10:90	Flavanone 39	1.3	1.4
		Ibuprofen 23	1.3	1.4
		Indooprofen 22	1.3	1.75
		Atenolol 3	1.3	1.45
		Aminoglutethimide 33	1.59	1
		Naftopidil 8	1.27	1.5
		1-Indanol 49	1.1	<1
		Pindolol 7	1.4	1.9
		Acebutolol 6	1.26	1.3
		Normatenphrine 30	1.3	1.27
		4-hydroxymandelic acid 44	1.34	1.6
	ACN:water 5:95	Atenolol 3	1.2	1.17
		Aminoglutethimide 33	1.4	1.6
		Ampicillin 25	1.3	1.2
		Pindolol 7	1.3	1.6

**Table 4:** Chromatographic parameters, separation, and resolution factors for the significantly resolved compounds on D3 (encapsulated) column, using reversed mobile phases, flow rate: 1 µL/min.

Column D3				
Phase	Mobile Phase	Drug	Separation Factor (α)	Resolution (Rs)
Reversed Phase	Methanol:water 40:60	Hexaconazole	1.14	1
		Aminoglutethimide 33	1.4	<1
	Methanol:water 5:95	O-methoxy mandelic acid 43	1.6	1.5
		Aminoglutethimide 33	1.4	1.8
	Methanol:water 3:97, 1% TFA	Metoprolol 5	1.4	1.3
		Atenolol 3	1.17	<1
	ACN:water 35:65	Fluribiprofen 21	1.25	1.5
		Propranolol 4	1.1	1.1
		Flavanone 39	1.2	1.44
		Ibuprofen 23	1.8	2.1
		Pindolol 7	1.3	1.5
		Naftopidil 8	1.3	1.23
		Normatenphrine 30	1.16	1.25
	ACN:water 20:80	Flavanone 39	1.2	1.3
		Atenolol 3	1.1	<1
		Tocainide 11	1.9	1
		pindolol 7	1.17	1.09
		Acebutolol 6	1.1	<1
		1-Indanol 49	1.36	1.55
		4-hydroxymandelic acid 43	1.3	1.55
		Cizoliritin carbinol 19	1.3	1.3
		Atenolol 3	1.14	<1
		Ibuprofen 23	1.3	1.4
		Naftopidil 8	1.3	1.55
		Ampicillin 25	1.3	1.45
		Pindolol 7	1.3	2
		Acebutolol 6	1.3	1.6
	ACN:water 5:95	Carprofen 15	1.2	1.38
		Metoprolol 5	1.1	<1
		1-In danol 49	1.36	1.64
		Normatenphrine 30	1.3	1.3
		4-hydroxymandelic acid 43	1.3	1.3
		Cizoliritin carbinol 19	1.29	1.5
		Ibuprofen 23	1.2	1.6
		Pindolol 7	1.28	1.3
	ACN:water 5:95, 1% TFA	Thalidomide 40	1.29	1.27
		Ampicillin 25	1.3	1.3
		Normatenphrine 30	1.4	1.4

## Data Availability

Data is contained within the article or Supplementary Materials.

## Supplementary Materials

The following are available online: Figure S1: Chemical structures of the tested racemic compounds, Figure S2: Nano-lc separation of tyrosin 35 (a), phenylalanine 36 (b), o-methoxy mandilic acid 43 (c) and 4-hydroxy-3-methoxymandelic acid 44 (d), on blank capillary column (150 µm ID, 25 cm length). Mobile phase: methanol/water 40:60 *v/v*, UV: 219 nm, flow rate: 1 µL/mL.
