# Supplementary material for: Daptomycin: A Novel Macrocyclic Antibiotic as a Chiral Selector in an Organic Polymer Monolithic Capillary for the Enantioselective Analysis of a Set of Pharmaceuticals
Source: Molecules. 2021 Jun 9;26(12):3527. doi: 10.3390/molecules26123527 (PMC8227699; doi:10.3390/molecules26123527)
Supplement: Supplementary file 1 [file molecules-26-03527-s001.zip › molecules-1244961-supplementary.pdf]

molecules

Supplementary Materials

Daptomycin: A Novel Macrocyclic Antibiotic as a Chiral Selector in an Organic Polymer Monolithic Capillary for the Enantioselective Analysis of a Set of Pharmaceuticals

Ali Fouad [1,2], Adel A. Marzouk [2], Montaser Sh. A. Shaykoon [2], Samy M. Ibrahim [3], Sobhy M. El-Adl [3] and Ashraf Ghanem [1,*]

1. Chirality Program, Faculty of Science and Technology, University of Canberra, Bruce, Australian Capital Territory (ACT) 2601, Australia. alifouad247@gmail.com (A.F.) ashraf.ghanem@canberra.edu.au (A.G.)
[2] Pharmaceutical Chemistry Department, Faculty of Pharmacy, Al-Azhar University, Assiut 71524, Egypt; alifouad247@gmail.com (A.F.); Adelmarzouk77@gmail.com (A.A.M.); monoceutical@yahoo.com (M.S.A.S.)
[3] Pharmaceutical Chemistry Department, Faculty of Pharmacy, Zagazig University, Zagazig, 44519, Egypt; drsamy2010@hotmail.com (S.M.I.);sobhyeladl@yahoo.com (S.M.E.-A.)
* Correspondence: ashraf.ghanem@canberra.edu.au

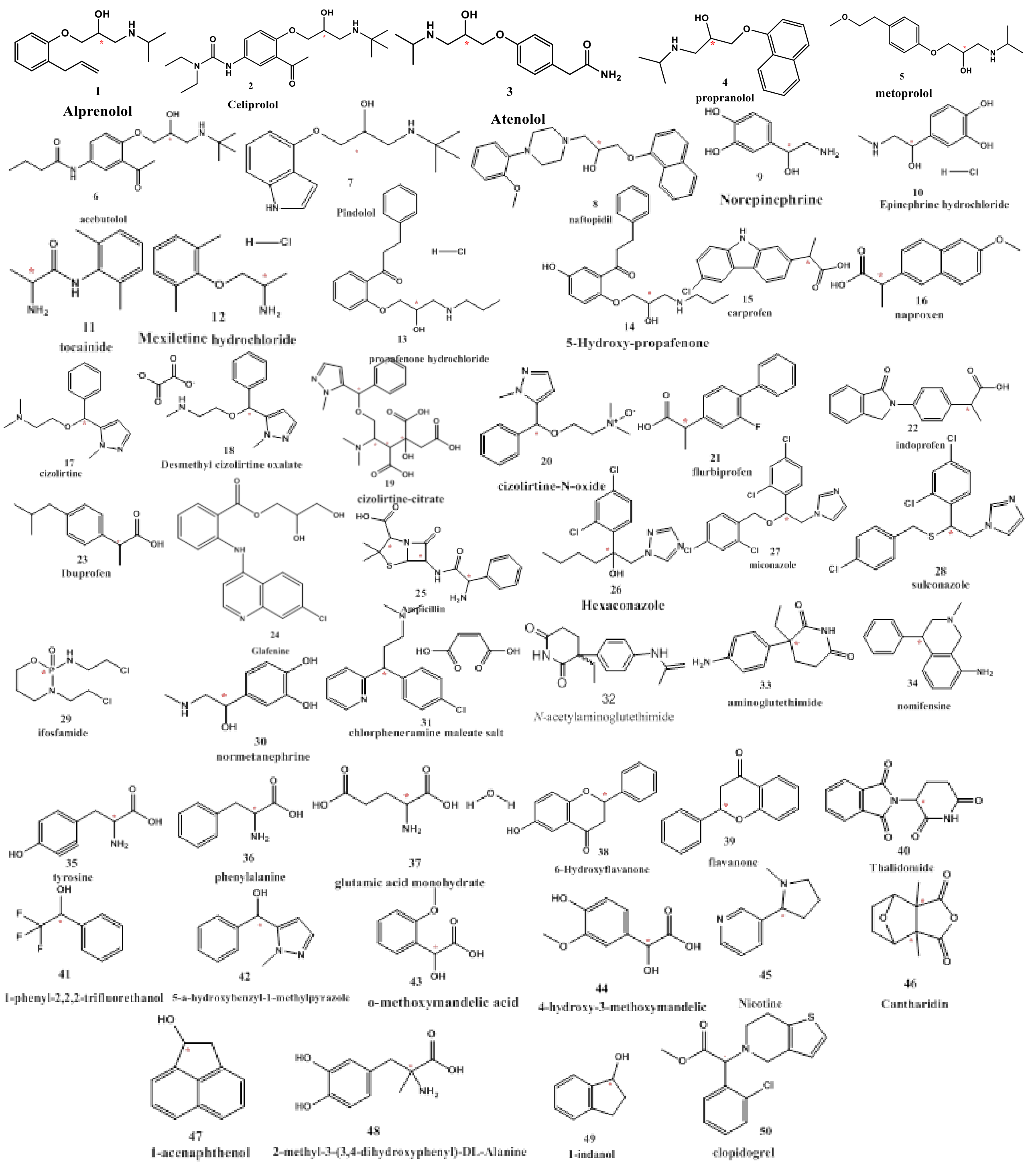

Figure 1. Chemical structures of the investigated racemates.

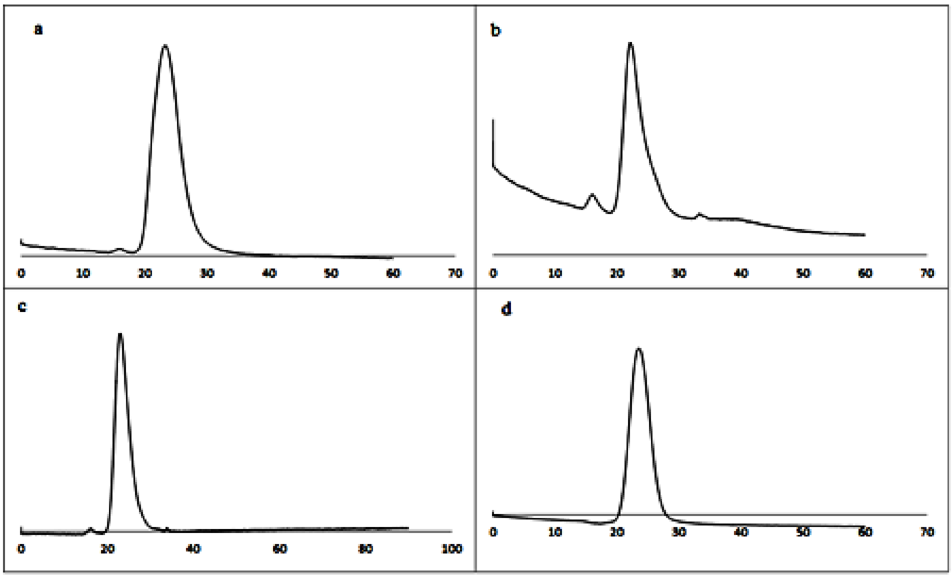

Figure 2. Nano-lc sep. of tyrosin 35 (a), phenylalanine 36(b), o-methoxy mandilic acid 43 (c) and 4-hydroxy-3-methoxy-mandelic acid 44 (d), on blank capillary column (150 μm ID, 25 cm length). Mobile phase: methanol/water 40:60 v/v,UV: 219 nm, flow rate: 1 μL/ml.
